# Latherin: A Surfactant Protein of Horse Sweat and Saliva

**DOI:** 10.1371/journal.pone.0005726

**Published:** 2009-05-29

**Authors:** Rhona E. McDonald, Rachel I. Fleming, John G. Beeley, Douglas L. Bovell, Jian R. Lu, Xiubo Zhao, Alan Cooper, Malcolm W. Kennedy

**Affiliations:** 1 Ecology and Evolutionary Biology, Faculty of Biomedical and Life Sciences, University of Glasgow, Glasgow, United Kingdom; 2 WestChem Department of Chemistry, University of Glasgow, Glasgow, United Kingdom; 3 Biological and Biomedical Sciences, Glasgow Caledonian University, Glasgow, United Kingdom; 4 Biological Physics Group, School of Physics & Astronomy, University of Manchester, Manchester, United Kingdom; Massachusetts Institute of Technology, United States of America

## Abstract

Horses are unusual in producing protein-rich sweat for thermoregulation, a major component of which is latherin, a highly surface-active, non-glycosylated protein. The amino acid sequence of latherin, determined from cDNA analysis, is highly conserved across four geographically dispersed equid species (horse, zebra, onager, ass), and is similar to a family of proteins only found previously in the oral cavity and associated tissues of mammals. Latherin produces a significant reduction in water surface tension at low concentrations (≤1 mg ml^−1^), and therefore probably acts as a wetting agent to facilitate evaporative cooling through a waterproofed pelt. Neutron reflection experiments indicate that this detergent-like activity is associated with the formation of a dense protein layer, about 10 Å thick, at the air-water interface. However, biophysical characterization (circular dichroism, differential scanning calorimetry) in solution shows that latherin behaves like a typical globular protein, although with unusual intrinsic fluorescence characteristics, suggesting that significant conformational change or unfolding of the protein is required for assembly of the air-water interfacial layer. RT-PCR screening revealed latherin transcripts in horse skin and salivary gland but in no other tissues. Recombinant latherin produced in bacteria was also found to be the target of IgE antibody from horse-allergic subjects. Equids therefore may have adapted an oral/salivary mucosal protein for two purposes peculiar to their lifestyle, namely their need for rapid and efficient heat dissipation and their specialisation for masticating and processing large quantities of dry food material.

## Introduction

Horses are flight animals that have a particular problem in dissipating heat efficiently during periods of sustained exercise. To do this they thermoregulate by producing copious amounts of sweat [1], a mechanism also used by humans but otherwise rare in mammals. Horses, however, have a thick, waterproofed, hairy pelt that would normally impede the rapid translocation of sweat water from the skin to the surface of the hair necessary for evaporative cooling. To solve this, horses appear to have evolved a surface-active, detergent-like protein that they release at unusually high concentrations in their sweat (human sweat is instead high in salt but low in protein). This protein, latherin, presumably acts by wetting the hairs to facilitate water flow for evaporation, the side effect of which is the lathering that is often observed on the pelts of sweating horses, especially where rubbing occurs. The best known surfactant proteins are those of the lung [2], [3], which also occur in other organs (ear, gut, reproductive tissues, synovium) [4]–[7]. About 90% of lung surfactant is lipid, the remainder comprising proteins of four kinds, ranging in activity from host defence via lipopolysaccharide and carbohydrate binding to reduction in surface tension to allow expansion of lung alveoli. Surface activity is mainly attributable to SP-B, which is a small, hydrophobic protein that interacts with phospholipids to produce a surface film [3]. Latherin, however, is non-glycosylated and there is no evidence that it is associated with lipids [8]. Latherin's biophysical activity must therefore be an intrinsic property of the protein itself. This is also a notable feature of the hydrophobins of fungi, where detailed structural studies have shown that surfactant activity and wetting ability is related to significant amphiphilicity of the native protein structure [9]–[11]. Many proteins can have surfactant effects, but this is usually confined to preparations of denatured protein [12], which, as we show, is not true of latherin.

Interest in biological surfactants has been steadily increasing since the 1960s when they first attracted attention as hydrocarbon dispersal agents with low toxicity and high biodegradability [13]. Recent studies have shown further potential for biological surfactants as antimicrobial activity or anti-adhesive agents against pathogens [14]. Such a dual function would make sense for latherin given that the pelt of a horse could be readily colonised by microorganisms potentially harmful to both skin and the hair itself, particularly following saturation sweating that would provide ample resources for the proliferation of microorganisms.

We report here on biophysical and molecular characterization of surfactant-related properties of recombinant latherin, including the cloning of cDNAs encoding latherin from several species of equid, and show that the recombinant protein possesses strong surfactant activity associated with self-assembly of an interfacial surface layer. We further show that latherin is also produced in horse salivary glands, which is consistent with their specialisation as animals needing to masticate and process large quantities of dry food material. So, equids may have adapted an oral/salivary protein for two purposes peculiar to their lifestyle, and it may be key to their ability to sustain high levels of exercise for long periods of time. Latherin, therefore, provides insight into an unusual specialisation of a large mammal and also how proteins on their own can act as surfactants in their native folded state.

## Results

cDNA encoding the complete precursor protein of horse (*Equus caballus*) latherin was obtained by RT-PCR and 5′- and 3-RACE procedures using oligonucleotide primers based on the amino acid sequences of tryptic fragments derived by Edman degradation of latherin obtained directly from sweat-derived protein, purified as previously described [8], and mRNA obtained from skin samples. Recombinant latherin (rlatherin) was produced without its hydrophobic secretory signal peptide and purified in high yield by over-expression in *E. coli*, and found to behave in solution as a well-folded globular protein, yet with significant surfactant properties, as follows.

### Sequence analysis – similarities and peculiarities

**Figure 1:**
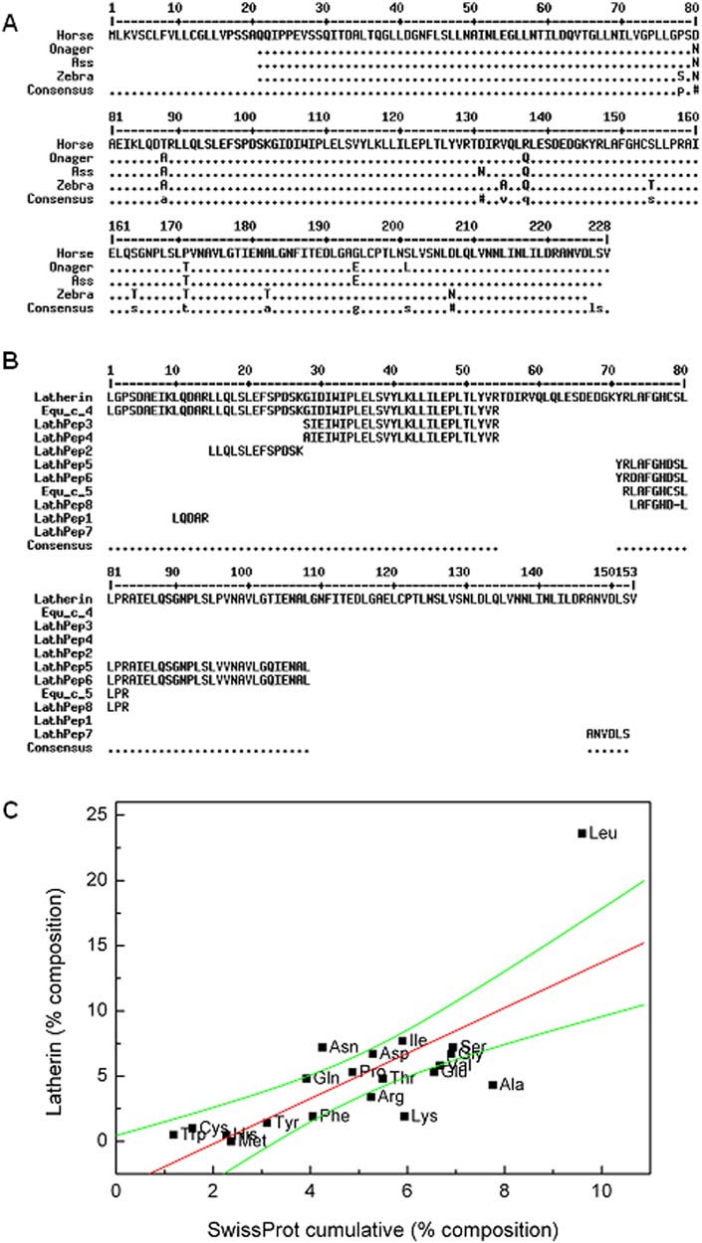
Analysis of latherin amino acid sequences. (A) Alignment of the amino acid sequences of latherin from domestic horses (*Equus caballus*), Persian Onager (*Equus hemionus*), Damara Zebra (*Equus burchellii antiquorum*) and Ass (*Equus asinus*). Most of the substitutions between the proteins are infrequently exchanged according to established evolutionary substitution matrices [52], [53], except for an exchange of aspartic acid (D) and asparagine (N), as indicated by the symbol #. (B) Alignment of the amino acid sequence of *Equus caballus* latherin predicted from cDNA, beginning at the first amino acid position for which there was a matching peptide, with the new peptide sequences (LathPep1 to LathPep8) and the peptide fragments previously ascribed to separate allergens Equ c 4 and Equ c 5 [16]. (C) Amino acid composition of the mature form of latherin obtained from its cDNA sequence, showing that the protein is unusually enriched in leucines. The percent composition of each amino acid in latherin is plotted against the cumulative composition of all proteins entered into Swiss-Prot (http://www.expasy.ch/sprot/relnotes/relstat.html). The red line is a simple regression line, and the green lines delimit the 95% confidence intervals.

Latherin from horse (*E. caballus*) comprises a 228-amino acid protein (Figure 1A), the first N-terminal 20 of which are predicted by SignalP [15] to be a secretory signal peptide expected to be removed post-translationally. The sequences of all the tryptic peptides obtained from sweat-derived latherin appear in the sequence encoded by the cDNA, as do those of horse allergens originally designated Equ c 4 and Equ c 5 [16] (Figure 1B). The allergen peptides therefore derive from latherin, although they may occur as separate entities through some degree of specific cleavage or degradation of the protein in horse dander. Similar procedures were used to obtain partial cDNAs encoding latherin of three other members of the Equidae, namely the Damara or Chapman Zebra (*Equus burchellii antiquorum*), Persian Onager (*Equus hemionus onager*), and an Ass (*Equus asinus asinus*). The amino acid sequences were very similar, with only a few amino acid differences (Figure 1A).

An analysis of the amino acid composition of the putative processed, mature, protein shows that latherin is slightly deficient in lysine and alanine, but remarkably enriched in leucine (23.6% for latherin versus 9.6% for the SwissProt cumulative average for all known proteins in that database; see Figure 1C).

Latherin's amino acid sequence allies it with a large family of proteins that includes the palate, lung and nasal epithelium (PLUNC) proteins, parotid secretory protein (PSP), lipopolysaccharide binding protein (LBP), and bactericidal/permeability-increasing protein (BPI) (see ref. [17] for a phylogenetic analysis). Both LBP and BPI bind bacterial lipopolysaccharide, and are therefore considered part of the innate immune system [18]–[20]. PLUNC proteins are currently of unknown function, but it is argued that they too are associated with innate immunity, specialized for action at mucosal surfaces [21]–[23], although formal demonstration of such activity is awaited. The proteins most closely related to latherin are the breast cancer and salivary gland-expressed (BASE) proteins (encoded by a defective gene in humans; [24]). Most of the PLUNC/BASE family do not have the high content of leucines noted above for latherin, with a few exceptions (e.g. human PLUNC Swiss-Prot Q9NP55; Leu 23%). Further confirmation of latherin's membership of the larger PLUNC family comes from the conserved position of its cysteines in amino acid sequence alignments with all members of that family. Moreover, an analysis of horse genomic DNA by PCR, using oligonucleotide primers flanking introns predicted from the positions of those in PLUNC genes, revealed that all the intron positions known in the latter are preserved in the latherin gene (not shown) [25], [26]. No definitive structural information is currently available for any PLUNC or BASE protein, although their probable similarity to the structures of BPI and LBP has been noted [17], [27]

### Structural characteristics

**Figure 2:**
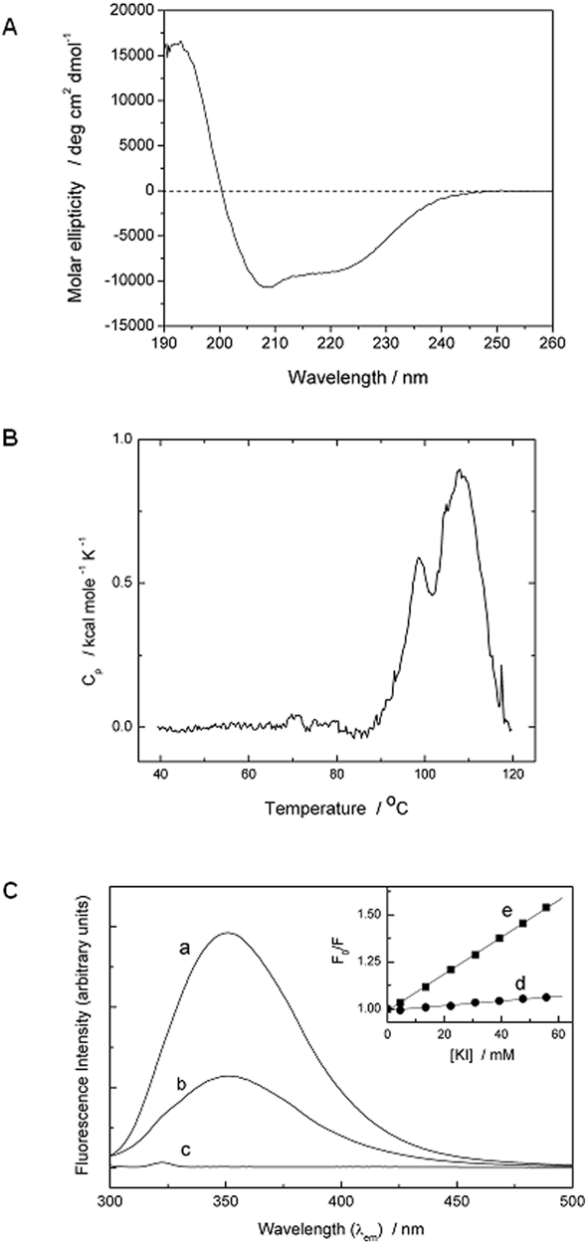
Biophysical analysis of recombinant latherin. (A) Circular dichroism spectrum of recombinant latherin recorded at 20°C using a 0.02 cm pathlength quartz cell, and a protein concentration of 1.3 mg ml^−1^. Spectrum analysis using the Selcon3 algorithm provided an estimated content of 30% α-helix, 18% β-sheet/extended structure, 22% β-turn and 29% other structure. (B) Differential scanning calorimetry demonstrating the thermal stability of recombinant latherin in solution. The sharp endothermic peak, with a T_m_ of approximately 105°C, is typical for irreversible unfolding of a globular protein. (C) Intrinsic tryptophan fluorescence (λ_ex_ = 290 nm) of recombinant latherin (5 µM) in (a) PBS and (b) 6 M GdnHCl/PBS, 25°C. The solvent baseline for GdnHCl/PBS (c) shows only the minor water Raman peak at around 323 nm. Inset: Example of the Stern-Volmer plot for iodide quenching of fluorescence at 350 nm for (d) latherin and (e) N-acetyl-Trp-amide (NATA), as control. See also Table 1 for quenching analysis.

Circular dichroism (CD) analysis of rlatherin indicated a mixture of α-helix and β-strand/extended secondary structure (Figure 2A), and spectrum analysis algorithms provided an estimated content of 30% α-helix, 18% β-sheet/extended structure, 22% β-turn and 29% other structure. Secondary structure prediction programs (e.g. PredictProtein, Jpred and SSPro4.5) all provided estimates of approximately 30% α-helix and slightly less β/extended structure. Consistently , all of these programs anticipate N- and C- terminal α-helix-rich regions flanking a central region of β/extended structure (see Figure S1) for an illustrative example). Differential scanning calorimetry (DSC) experiments showed that rlatherin has good thermal stability in solution, with no apparent thermally-induced changes below 85°C, and that it undergoes endothermic unfolding at higher temperatures (T_m_≈105°C, see Figure 2B). This is typical behaviour for the irreversible thermal unfolding of an extra-cellular protein in aqueous solution. Fluorescence emission from ANS (8-anilino-1-naphthalenesulfonic acid), a common probe for hydrophobic patches or (partially) unfolded protein, was unchanged in the presence of rlatherin (not shown). All these properties are characteristic of a stable, folded, soluble globular protein, and this is confirmed by our preliminary high-resolution protein NMR data (not shown) demonstrating that rlatherin is well folded in solution.

### Protein intrinsic fluorescence

The molecular environment of latherin's single conserved tryptophan residue (Trp-107) was examined by fluorescence emission and quenching experiments. Intrinsic fluorescence (Figure 2C) shows a peak emission of the recombinant protein at 350 nm when excited at 290 nm. This is somewhat more red-shifted than normally observed for globular proteins [28], [29], and shows a decrease in intensity but no wavelength shift under denaturing conditions (6 M Gdn HCl), similar to that of an indole group fully exposed to solvent water.. It is therefore likely that Trp107 is exposed to solvent water.

This degree of exposure is unusual for tryptophan side chains, but is commonly found in membrane-inserted proteins, and has been found as so-called ‘sticky fingers’ in a number of soluble proteins (reviewed in ref. [30]). In the latter case, such unusually positioned side chains could be involved in protein∶protein or protein∶membrane interactions. In the case of latherin, an exposed Trp side chain might be involved in protein surface hydrophobicity related to surfactant activity. Apolar surface patches in hydrophobins are considered to be key to their surface activity and also their self-assembly into higher order complexes that enhance surface activity [10], [11], though in rlatherin we find no evidence of hydrophobic patches detectable by ANS binding (see above).

However, an alternative explanation of the unusual intrinsic fluorescence could be that the Trp side chain of latherin is located away from bulk water, but in an unusually polar environment within the protein structure. We have attempted to clarify this using standard fluorescence quenching probes (iodide, acrylamide, succinimide), but with somewhat ambiguous results (see Table 1). Intrinsic Trp fluorescence was reduced in the presence of quenchers, and all three probes gave linear Stern-Volmer plots (Figure 2C inset) symptomatic of collisional quenching for a single Trp-containing protein [28], [31]; quenching parameters (K_SV_) are given in Table 1. Paradoxically for a putatively exposed tryptophan side-chain, quenching was lowest with iodide – normally considered to be most effective in quenching exposed groups, but likely to be strongly influenced by local electrostatic effects (refs. [28], [31] and refs therein). Neutral quenchers (acrylamide, succinimide) were much more effective, with relatively high K_SV_ values comparable to those previously observed for proteins with relatively exposed Trp residues [28], [31]. Interestingly, these quenching parameters were not significantly affected by either thermal or chemical denaturation of the protein (see Table 1). This suggests that the latherin Trp may well be in an exposed, polar environment, but protected from collisional iodide quenching by surrounding negative charges or other local sequence-specific effects. It has recently been shown that tryptophan fluorescence and its quenching can be significantly affected by local amino acids, even in small model peptides in strongly denaturing environments [32], [33]. Significantly, and in contrast to previous assumptions, this work has shown that quenching by both acrylamide and iodide is highly variable and sequence dependent, with iodide quenching in particular being significantly reduced in the presence of nearby negatively-charged residues. Latherin is an acidic protein (estimated pI = 4.11) and will carry a net negative charge of around −14 at neutral pH, and this is likely to inhibit collision with I^−^ ions by electrostatic repulsion. Moreover, Trp-107 is adjacent in sequence to an aspartate residue (Asp-105), a juxtaposition that has been shown to inhibit iodide quenching in model peptides [33]. Consequently, although we know nothing yet about the detailed molecular structure of latherin in solution, it appears that the single conserved tryptophan residue is located in a relatively exposed location, possibly poised and ready to facilitate “sticky-finger-like” unfolding of the protein at hydrophobic interfaces.

**Table 1:** Comparison of Stern-Volmer coefficients for quenching of Trp fluorescence by assorted quenchers.

	K_SV_/M^−1^		
	Iodide	Acrylamide	Succinimide
N-acetyl-trptophan-amide	9.75±0.1 (6.8–8.9)^a^	17.6±0.2 (17.5)^b^ (17–20)^a^	10.9±0.2 (11)^c^
Latherin	1.25±0.1	8.2±0.2	4.7±0.1
Latherin in 6 M GdnHCl	1.9±0.5	7.6±0.2	*nd*
Thermally-denatured latherin^d^	1.3±0.2	*nd*	*nd*

(literature values for N-acetyl-tryptophan-amide in parentheses).

a in urea or GdnHCl, from [33].

b from [29].

c from [31].

d irreversibly denatured sample taken from DSC after repeat scans to 110°C.

*nd* = not determined.

### Surfactant properties - surface tension

**Figure 3:**
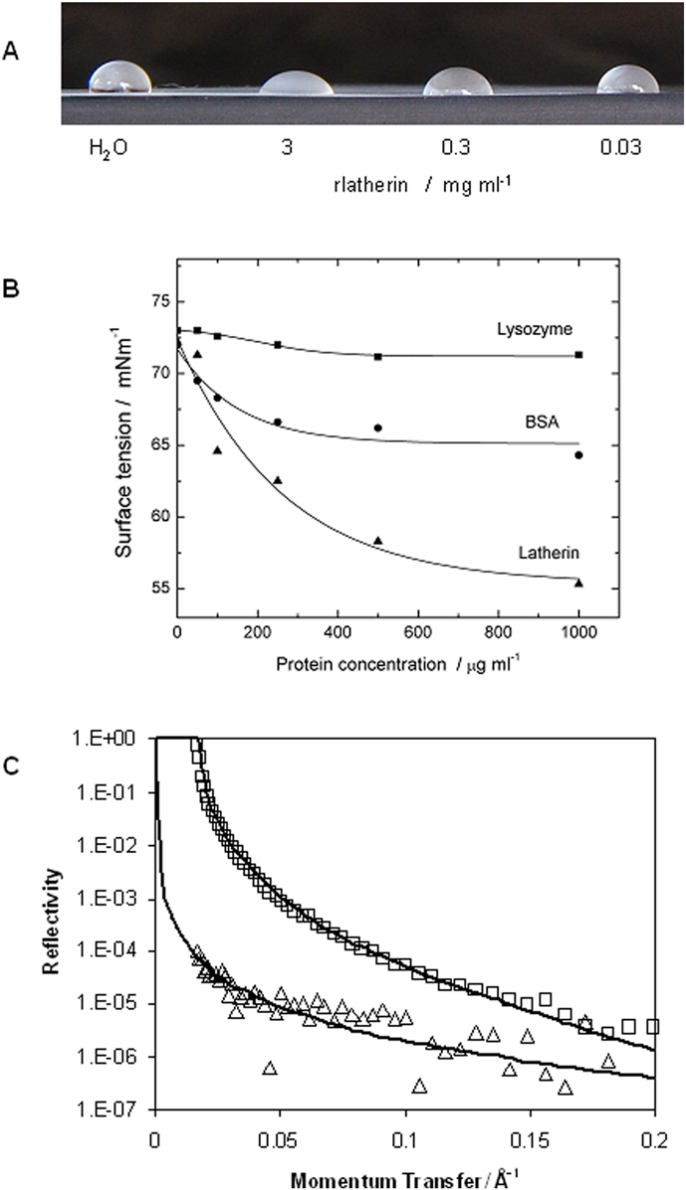
Surface activity of latherin. (A) 20 µl drops of water or solutions containing different concentrations of recombinant latherin on a waxy surface (®Nescofilm). (B) The surface tension of solutions containing either chicken egg lysozyme, bovine serum albumin (BSA) or recombinant latherin at the concentrations indicated, measured using the de Nuoy ring method. The fitted lines are for guidance only. (C) Neutron reflectivity profiles for 0.5 mg ml^−1^ latherin dissolved in D_2_O (□) or non-reflective water (Δ), pH 7, respectively. The solid lines are the theoretical fits to a single-layer model for adsorption of latherin at the air-water interface with parameters given in Table 2.

In previous work [8] the surfactant properties of natural latherin had been characterized using a capillary method, and we found that recombinant latherin was similarly active; at approximately 1 mg ml^−1^, rlatherin substantially decreased the contact angle of water droplets on a hydrophobic surface (Figure 3A). Using the more quantitative Du Nouy ring method, the protein was found to be substantially more active at reducing the surface tension of water than either lysozyme (negligible effect) or bovine serum albumin (which presumably owes its activity to contamination with lipid or the presence of surface-proximal hydrophobic ligand binding sites) on a weight basis (Figure 3B). At protein concentrations above 0.1 mg ml^−1^ the surface tension of latherin drops quickly from the pure water value of around 74 mN m^−1^, reaching 56 mN m^−1^ at 1 mg ml^−1^, a concentration approximating that of latherin in horse sweat [8]. This reduction in surface tension is typical of detergent-like behaviour, and explains the ease with which horse sweat and/or dilute latherin solutions foam and wet hydrophobic surfaces.

Surface wetting is likely to be accompanied by adsorption of surfactant protein molecules onto a hydrophobic surface. This was previously indicated by contact angle experiments with natural latherin showing that a hydrophobic surface pre-exposed to natural latherin was wettable, presumably because of adsorbed protein [8]. We have confirmed this here for recombinant latherin, using Nescofilm® as a model hydrophobic/waxy surface, showing that rlatherin absorbs to this surface, unlike a control protein (hen egg white lysozyme) (see Figure S2, for details). Interestingly, absorbed rlatherin could also be removed by gentle washing with water prior to protein staining. This latter feature is presumably of functional significance, since permanent wetting would compromise the natural water-repellent properties of the oily pelt and provide a nutritive source for microorganisms.

### Surfactant properties - neutron reflection

The strong surfactant activity of rlatherin, manifest in its ability dramatically to reduce the surface tension of water at low concentrations, is unusual for a structurally intact soluble protein and implies that it must undergo significant adsorption at the air-water interface of aqueous solutions. This is confirmed by neutron reflection experiments (Figure 3C) showing that the surface of dilute aqueous latherin solution contains adsorbed protein, best modelled as a single layer approximately 10 Å thick. Detailed parameters for 0.5 mg ml^−1^ solutions are given in Table 2. Pure D_2_O has a scattering length density (SLD) of 6.35×10^−6^ Å^−2^, whereas NRW (null reflection water) is a D_2_O/H_2_O mixture with zero SLD. By comparing results in these two solvents, information on how the layer is packed and its location relative to the water surface (above or below the interface) is inferred. The adsorption of latherin in D_2_O was best modelled as a single protein layer fully immersed in the interface, with layer thickness of 10 Å. The calculated theoretical SLD of the pure protein in D_2_O would be 2.87×10^−6^, compared to the experimentally obtained value of 4×10^−6^. The volume fraction of latherin in the layer may thereby be estimated to be 0.67. The estimated surface excess is 0.87 mg m^−2^ with an area per molecule of 4350 Å^2^. Similar results were obtained for latherin both at lower concentration (0.1 mg ml^−1^) and in NRW (Table 2).

**Table 2:** Best fit neutron reflection parameters for a single-layer surface adsorption of latherin from 0.5 mg ml^−1^ solution in D_2_O or null-relecting water (NRW).

Solvent	Layer Thickness/Å	ρ_obs_ a/10^−6^	ρ_p_ b/10^−6^	Volume Fraction^c^	Surface Excess/mg m^−2^	Area per Molecule/Å^2^
D_2_O	10	4	2.87	0.68	0.872	4355
NRW	10	1.2	1.75	0.69	0.885	4289

a Neutron scattering length density from experimental fits.

b Calculated from amino acid sequence data, assuming appropriate H/D exchange.

c Calculated using ρ_obs_ = φ_p_ρ_p_+φ_w_ρ_w_.

This interfacial layer seems surprisingly thin for a 23 kDa protein layer in equilibrium with the bulk solution where, judging from CD and DSC observations, latherin behaves as a folded globular protein. We have recently used infrared reflectance to demonstrate the presence of both intact alpha-helix and beta-structures for a different surfactant protein that also appears to be a folded globular protein in bulk solution [34]. The 10 Å layer thickness for latherin is similar to that observed for the adsorption of β-hairpin peptides on the surface of water, using the same neutron reflection methods [35]. This suggests that, despite its apparent stability in bulk solution, latherin must undergo significant conformational change and/or partial unfolding during incorporation into the interfacial layer. This makes sense in functional terms. Soluble surfactant proteins need to reconcile surfactant activity at the site of operation with the requirement for solubility and lack of aggregation during synthesis, storage, and transport stages. Conventional (small molecule) detergents are amphiphilic and can form soluble aggregates (micelles) in solution that can readily dissociate to form surface monolayers when required. This would be a difficult strategy for protein surfactants, since the large exposed hydrophobic surfaces required at interfaces would lead to significant solubility and aggregation problems in bulk solution. Latherin may overcome this by folding as a normal globular protein in solution, with a predominantly polar outer surface giving aqueous solubility, yet with the ability to unfold at a hydrophobic/air-water interfaces to expose previously buried non-polar residues to the surface. Note that this does not necessarily require complete denaturation of the protein but simply partial unfolding and/or domain rearrangement, as observed elsewhere [34].The unusually high leucine content of latherin, atypical of soluble globular proteins, may be related to this proposed surfactant mechanism.

### Locations of latherin gene transcription and protein

**Figure 4:**
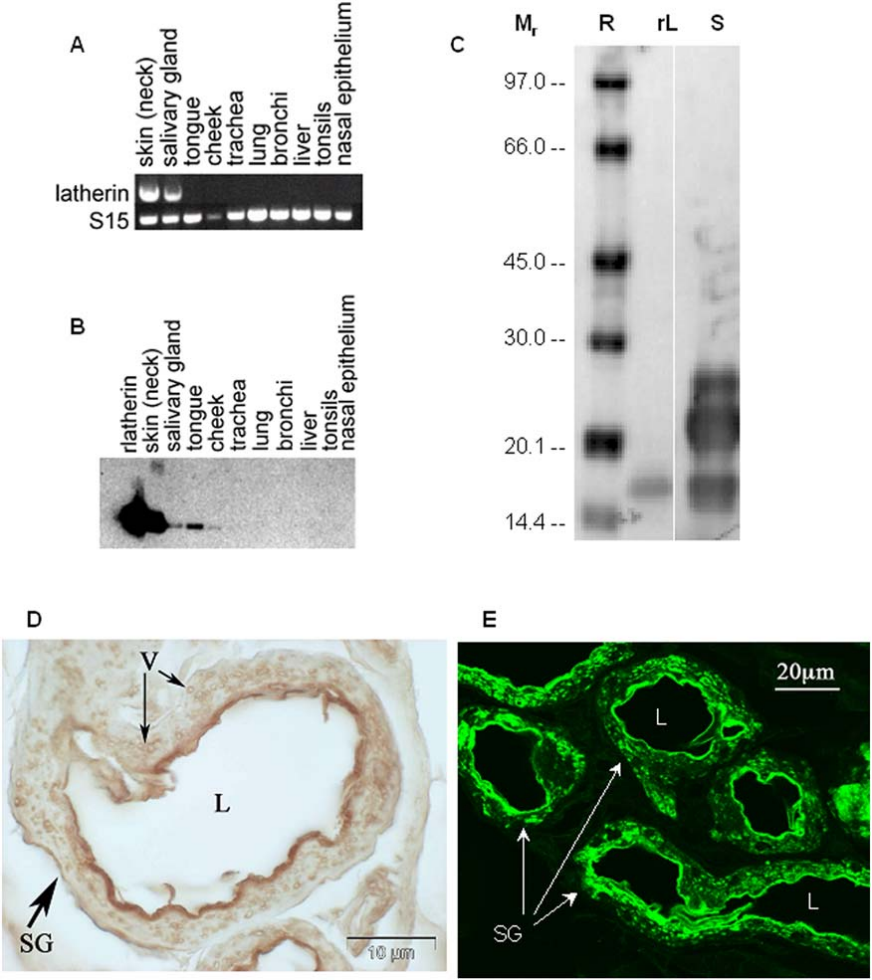
Tissue sites of latherin gene transcription and immunological detection of latherin protein. (A) RT-PCR using gene-specific oligonucleotide primers to detect latherin-encoding transcripts in a range of horse tissues; the ribosomal subunit protein S15 was used as a control. (B) Immunoblot using latherin-specific antibody to detect latherin protein in tissue samples. (C) Immunoblot showing IgE antibody reactivity of serum from a horse-allergic patient binding to recombinant latherin (rL) and horse sweat (S). The protein visible at ∼M_r_ 21,000 is probably the major allergen in horse sweat, Equ c 1. Five out of nine patients with detectable IgE antibody reactions to horse sweat exhibited IgE antibody to recombinant latherin. Sera from non-allergic individuals did not react to any components in horse sweat or the recombinant latherin. R – reference proteins with M_r_ expressed in kDa. (D) Immunoperoxidase labelling of a section of normal equine skin showing a dense dark band (brown) of staining proximal to the apical membrane of the sweat gland secretory epithelial cells; cell surface associated latherin is likely to have been lost during the preparation process. Staining is also apparent in intracellular vesicles. SG = sweat gland, L = lumen, V = vesicles. (E) Immuno-fluorescent staining for latherin in equine sweat glands. Bright staining indicates the presence of latherin inside the secretory cells of the gland. (SG = sweat glands, L = lumen).

A range of tissues was sampled from a horse, with mRNA prepared for detection of latherin transcripts by RT-PCR, and protein extracted for immunodetection using a rabbit antibody to recombinant latherin (rlatherin). As shown in Figure 4A, latherin transcripts were only detectable in skin and (submaxillary) salivary gland, and the protein was only detectable from skin, salivary gland, tongue and cheek (Figure 4B). In the case of the latter two sites, the tissue samples included epithelium, in which no transcripts were detected in separate experiments, so it is likely that the protein detected there was derived from contaminating saliva. In a more limited screen, we found, as expected, that the gene for the major allergen of horses, Equ c 1, a lipocalin of unknown function though may be a pheromone carrier [36]–[38], was also transcribed in skin and submaxillary gland tissue. Immunohistochemistry and immunofluorescence showed that latherin is present within the cells of the fundus region of the sweat gland (Figure 4D & E), which is the region with cells containing apparent storage vesicles/granules that are lost during copious sweating [39]. Figures 4D & E also show localisation of latherin to vesicles/granules within the cells of the secretory epithelium.

### Latherin as allergen

As mentioned above, the amino acid sequence of latherin encompasses two peptides that have previously been ascribed to the separately classified allergens Equ c 4 and Equ c 5 [16]. We obtained serum samples from eight horse-allergic patients and examined the antigen binding activity of their IgE antibody (see Figure 4C for a typical result). This showed that patient IgE antibody bound to several components of horse sweat, the major band being at approximately M_r_ 21,000, which would correspond to the major allergen of horses, Equ c 1 (which is also produced both in skin and saliva, [16], [36], [37]). The band at approximately M_r_ 17,000 co-migrates with recombinant latherin (rlatherin), which is also bound by the patients' IgE antibody. This immunoblot also shows the more rapid migration exhibited by latherin in SDS-PAGE than expected for its size, possibly due to its very high content of apolar amino acids and consequent more rapid migration caused by a high degree of binding by SDS detergent.

## Discussion

These biochemical and biophysical observations suggest the evolution of a dual role for latherin as both a sweat and a salivary protein. Its presence in equine sweat is consistent with a role in wetting hair and skin to allow rapid translocation and spreading of sweat water at the surface of the oily pelt for evaporative cooling. Interestingly, human skin secretions are also thought to have a surfactant-like role in enhancing the spreading of sweat water for cooling, though by a mechanism suited to an effectively hairless surface ([40], [41]). The deposition of large quantities of protein through and over the pelt of horses would seem to present the risk of providing a nutrient resource for microorganisms. It could therefore be that latherin's surface activity may also directly affect the surfaces of microbes or impede their adhesion and establishment on hair and skin. Latherin is distantly related to proteins that are directly anti-microbial [21], but we have been unable to detect any interaction between latherin and archetypical bacterial components such as lipopolysaccharide (not shown). A function for latherin in horse saliva is less obvious, but the presence of a surfactant protein there may assist mastication and processing of large quantities of relatively dry vegetable matter, a diet for which the equids as a group are specialised, unlike ruminants (cows, sheep, etc.).

The possibility that latherin's surface-activity presents a danger to mammalian cells, however, needs to be addressed, as does the mechanism by which the intracellular membrane systems involved in synthesis, processing and export of the protein are protected from damage. A simple small molecule detergent would normally be extremely damaging to cellular membranes, but it is possible that proteins can be designed to perform detergent-like functions without endangering cell membranes. An example of this could be the strong surface activity of the proteinaceous foams in which certain tropical frogs lay their eggs, which may also have anti-microbial propensities, but are nonetheless compatible with naked eggs and spermatozoa [42], [43]. It is possible that latherin is specially packaged by the synthesising cells before release, and our immunohistological observations on horse skin using anti-latherin antibody indicate that the protein is localised to the protein granules of the cells in the fundus region of the sweat gland, which are known to be exocytosed directly upon sweating [39]. At a more fundamental level, the mechanism by which small-molecule detergents solubilise lipid bilayer membranes is likely to be inapplicable to larger, surfactant proteins. Conventional (synthetic) detergents such as SDS, Triton, etc., exert their effects by formation of micelles in aqueous solution (into which lipids can be extracted) as well as by monolayer formation at the air-water interface (to reduce surface tension). In contrast, although specific surfactant proteins do partition into the air-water interface [42], their size and (presumably) structure precludes any equivalent of micelle formation in the bulk solution, therefore minimizing disruption of lipid bilayers.

There are now several different classes of protein that exhibit biologically relevant, intrinsic surface-activity, including latherin, the hydrophobins and frog foam nest foam proteins (ranaspumins). It would be valuable to know what structural characteristics separate or unify these proteins so as to extend our understanding of how proteins can do what they do. In the case of latherin, we are fortunate that it can be produced in soluble recombinant form in bacteria, and we have already obtained high quality protein NMR spectra as a preliminary to solving its solution structure (R.E. MacDonald, A. Cooper, B.O. Smith and M.W. Kennedy, unpublished). A structure for latherin may also improve our understanding of the PLUNC and BASE proteins, some of which may also possess intrinsic surface activity in order to operate in mucosal surfaces. Meanwhile, in the absence of indications that PLUNC family members are produced in mammalian skin, latherin may represent a remarkable adaptation of a salivary protein for heat dissipation from skin by horses and their ilk, and a readily available protein with which to study intrinsic surface activity of a naturally folded protein from a mammal.

## Materials and Methods

### Ethical statement

All tissue samples used in the study were collected from animals that died from, or were euthanised for, clinical causes entirely unconnected to the investigation, and with permission from the owners, and for which no formal ethical permission is required under UK Home Office regulations. Sweat was collected from racehorses by gentle use of an equine sweat collector in Scotland under permission of the owners and Glasgow University's Veterinary Ethics and Welfare Committee and in Trinidad under permission from the owners and the Chief Veterinary Officer of the Republic of Trinidad and Tobago.

### Biological samples

Skin samples from domestic horses (*Equus caballus*), a Damara Zebra (*Equus burchellii antiquorum*), and an Onager (*Equus hemionus*) were collected from recently deceased animals in the UK, together with an Ass (*Equus asinus*) from Muscat and Oman, and stored immediately in RNAlater (Ambion, Warrington, UK). Other tissues were collected from horses euthanized for clinical reasons unrelated to the study within 90 mins of death and stored in RNAlater at −20°C until further use. Sweat was collected from racehorses in Scotland and Trinidad.

### Latherin peptide sequencing, cDNA cloning and sequencing

Natural latherin was purified from horse sweat and tryptic peptide fragments were sequenced as described previously [8]. Specifically, portions of reduced and carboxymethylated latherin were dissolved in 0.1 M ammonium bicarbonate buffer, 0.1 mM CaCl_2_, and digestions were carried out with TLCK-trypsin (Sigma, Poole, Dorset, UK) or endoproteinase Lys-C (Boerhinger-Mannheim, Mannheim, Germany) at an enzyme∶substrate ratio of 1∶50 w/w at 37°C for 3 hr. Substantial precipitation occurred in both enzymic digests, and the insoluble peptides were separated by centrifugation. Peptide fractions were lyophilised repeatedly to remove ammonium bicarbonate. The soluble (S) peptides were dissolved in 0.1% w/v aqueous trifluoroacetic acid (TFA). The insoluble (I) peptide fractions dissolved readily in acetonitrile which was then diluted 1∶1 with 0.1% aqueous TFA giving a clear solution. Aliquots of the S and I peptide mixtures were fractionated by reversed phase chromatography on a silica-C8 reversed phase column (Waters, Elstree, Hertfordshire, UK) using a solvent gradient of increasing acetonitrile concentration containing 0.1% TFA. Peptide fractions were pooled and aliquots hydrolysed and subjected to amino acid analysis [8]. Samples of peptides were sent for analysis by liquid phase automated Edman degradation at the Department of Biochemistry, University of Aberdeen. These sequences (Figure 1B and Table S1) were used to design oligonucleotide primers (Table S2) for the isolation of cDNA encoding the protein. RNA was isolated from skin samples using TriZol reagent (Invitrogen Life Technologies, Paisley, UK) and mRNA reverse transcribed to cDNA using a 3′ and 5′ RACE kit (Clontech, Basingstoke, Hampshire, UK). Conditions for the 3′ RACE PCR reactions were 94°C for 3 mins, followed by 30 cycles of 55°C (30 s), 72°C (60 s) and 94°C (30 s) using a Biometra (Goettingen Germany) Tgradient® thermal cycler. PCR products were inserted into the TA sequencing kit for sequencing (Invitrogen). This procedure provided cDNA sequence for horse latherin, and the encoded protein contained all the peptide sequences obtained from sweat-derived latherin (Figure 1B and Table S1). Similar procedures were used to obtain partial cDNA sequences from the other equid species, using additional primer sets designed to overlap those of the original primer positions to ensure that the final sequences were fully correct for each species. All the sequences have been deposited in GenBank under the accession codes AF491288, AY226145, AY881016 and AY881017.

### Recombinant latherin protein

cDNA encoding latherin, excluding the leader peptide, was amplified by PCR using appropriate oligonucleotide primers (Table S2), and directionally inserted into NcoI, XhoI sites of the pET32a expression vector, and the resulting ‘LathpET32’ plasmid was transformed into BL21(DE3)pLysS cells. Individual transformant clones were grown overnight at 37°C in 10 ml of Luria-Bertani (LB) medium containing 50 µg ml^−1^ carbenicillin and 34 µg ml^−1^ chloroamphenicol, and used to seed 500 ml flasks of LB medium supplemented with the appropriate antibiotics. The cultures were grown until an OD_600_ of 0.6–0.8 was reached, at which time the cultures were induced with a final concentration of 1 mM IPTG. These cultures were grown for a further 4 h, at which time cells were harvested by centrifugation and the cell pellet stored at −20°C. The fusion protein was purified by affinity chromatography with the HisBind resin and buffer kit, in accordance with the manufacturer's instructions (Novagen/Merck Chemicals, Nottingham, UK). The thioredoxin tag was cleaved by overnight incubation with recombinant enterokinase (Novagen), and the enzyme removed by passing through a column packed with EK Agarose (Novagen). The solution was diluted with binding buffer (20 mM Tris, 0.5 M NaCl, 5 mM imidazole, pH 7.8) and the thioredoxin tag removed by passing through a nickel affinity column. Recombinant latherin was further purified by gel filtration (Superdex 75, 16/60, 50 mM Tris, 0.25 M NaCl, pH 8.4; Amersham Pharmacia/GE Bioscience, Chalfont St Giles, Buckinghamshire, UK).

### Reverse Transcriptase PCR

mRNA was extracted from equine tissues using Trizol treated with DNAase I (Ambion) to remove genomic material. cDNA was synthesised using Superscript II reverse transcriptase (Invitrogen), using an oligo T primer. RNA complementary to the cDNA was removed using RNAase H (Invitrogen) prior to PCR. cDNA encoding either latherin or as control the ribosomal S15 protein were as listed in Table S2. Reactions comprised 0.5 µl cDNA, 1 µl each primer (100 pM), 1 µl dNTPs (40 mM), 5 µl polymerase buffer, 4 µl MgCl_2_ (25 mM), 2.5 U Taq polymerase (Promega, Southampton, UK) and sterile water to a final volume of 50 µl. The PCR conditions used were 94°C, 4 min; 94°C, 15 sec; 65°C, 30 sec 10 cycles decreasing annealing temp 1°C each time; 72°C, 30 sec, 94°C, 15 sec; 55°C, 30 sec 25 cycles; 72°C, 30 sec followed by a 5 min extension at 72°C.

### Immunohistochemistry and immunofluorescence

Skin samples were obtained from lateral neck skin of horses in the UK immediately after euthanasia for veterinary reasons not associated with skin complaints. The samples were immediately fixed in 10% neutral buffered formalin, dehydrated through a series of graded alcohols, cleared in Histoclear™ (Flowgen Bioscience, Nottingham, UK) and embedded in paraffin wax. Four-micron sections of equine skin containing sweat glands were mounted on 3-aminopropyl-triethoxy-silane (APES) treated slides. Sections were de-waxed, re-hydrated to phosphate buffered saline and endogenous peroxidase activity was blocked by treatment in 3% H_2_O_2_ for 10 min. High-pressure antigen retrieval, to unmask antigenic epitopes, was performed by immersing sections in pre-warmed EDTA buffer (EDTA 1 mM and Tris base 4.5 mM, pH 8) and heating them in a microwave pressure cooker for 5 min. The sections were allowed to cool before blocking with 20% horse serum in PBS for 30 min. Endogenous avidin and biotin sites were blocked using an Avidin Biotin Blocking kit (Vector Laboratories, Orton Southgate, Peterborough, UK) involving incubation with each blocker for 30 min. Sections were then incubated overnight at 4°C with rabbit-derived antibody against latherin. After washing in PBS, sections were treated using a Vectastain Elite ABC kit (Vector Laboratories). Briefly, they were incubated for 30 min in biotinylated secondary antibody (1∶50 horse anti rabbit) then washed and incubated for 30 min in avidin-biotin peroxidase complex (1∶50) before being washed and developed under the microscope using a DiaminoBenzidine/H_2_O_2_ solution kit (Vector Laboratories). Negative controls included omission of primary antibody. For immunofluorescence, sections were prepared as above, except that after incubation with the primary antibody the sections were further incubated at 37°C for 1 hour using an anti-rabbit fluorescein-labelled secondary antibody. Sections were subsequently washed in PBS for 10 minutes and then mounted with a glass coverslip using an aqueous mounting media (Dako Ltd, Ely, Cambridgeshire, UK). Sections were viewed on an Olympus BH2 light and fluorescence microscope and images acquired using ‘analySIS’ software (Soft Imaging Systems, GmbH, Germany), on a PC computer connected to a microscope-mounted Olympus BX50 digital camera.

### SDS-PAGE and immunoblotting

Protein samples were separated on Precast NuPage 4–12% Bis-Tris gels (Invitrogen) and visualised with Coomassie Blue. For immunoblots, separated proteins were electrophoretically transferred onto a nitrocellulose membrane (Hybond ECL, Amersham Pharmacia/GE Biotech) using a Criterion blotter (Bio-Rad Laboratories). The membrane was blocked (5% skimmed milk powder, 1% Tween 20 in PBS) for 1 h at room temperature, and then rinsed in the same buffer. The membrane was then incubated with primary antibody (rabbit anti-latherin antibody 1∶10,000 dilution) for 1 h. After washing, membrane strips were incubated with secondary antibody (goat anti-rabbit horseradish peroxidase 1∶10,000, Bio-Rad laboratories, Hemel Hempstead, Hertfordshire, UK) for 1 h. The membrane was washed in PBS/0.1% Tween 20 followed by a water wash. Enhanced chemiluminescence (ECL) reagents were used to detect specifically bound second layer antibodies according to the manufacturer's instructions. The bands were visualised either by exposure to X-ray film (Hyperfilm, Amersham Pharmacia) or in an image analysis system (Kodak, Hemel Hempstead, Hertfordshire, UK). Rabbit antiserum was raised against recombinant latherin by PTUBS, Pentlands Science Park, Bush Loan Midlothian. Human sera from horse allergic and negative control subjects were obtained from an anonymous collection provided by Dr C. McSharry, Faculty of Medicine, Glasgow University. Immunoblotting membrane strips contained 0.1 mg ml^−1^ recombinant latherin, horse sweat was diluted to 1∶20, test sera were diluted 1∶20 and incubated with the strips overnight at 4°C. Following overnight incubation, membranes were washed and probed with biotinylated anti-human IgE (5 µg ml^−1^) followed by horseradish peroxidase streptavidin (5 µg ml^−1^). ECL was used, as above, to visualise any antibody/allergen reactions.

### Surface tension measurements

Surface tension was measured manually using the Du Nouy ring method and a White torsion balance calibrated using ultrapure water (UHQ, Elgastat, Elga, UK). Experiments were carried out at room temperature and the platinum ring cleaned between readings by rinsing with UHQ water and flaming to remove residual deposits. Contact angles and wettability were examined using small droplets placed on a hydrophobic surface (Teflon tape or ®Nescofilm), photographed side-on using a digital camera.

### Circular dichroism

CD spectra were recorded at 20°C in a JASCO J-600 spectropolarimeter using a 0.02 cm pathlength quartz cells with a protein concentration of 1.3 mg ml^−1^, and the spectra analysed using the SELCON3 [44], [45] procedure to estimate secondary structure content. Secondary structure predictions were made using the PredictProtein, Jpred and SSPro v4.5 routines accessed through the ExPASY internet site (www.expasy.ch).

### Differential scanning calorimetry (DSC)

The thermal stability of latherin in solution was investigated using a MicroCal VP-DSC (MicroCal LLC, Northampton, MA, USA) at a scan rate of 60-°C hr^−1^. Samples were dissolved in phosphate buffer, pH 7, at a concentration of approximately 1 mg ml^−1^, and degassed briefly prior to loading in the DSC cell. Data were analyzed using standard MicroCal Origin instrumental software.

### Protein fluorescence

Intrinsic tryptophan fluorescence emission of latherin in solution (phosphate buffer, pH 7, 25°C) was measured using 1 cm quartz cuvettes in a Spex Fluoromax II spectrofluorimeter (HORIBA Jobin Yvon Ltd, Stanmore, Middlesex) with excitation wavelength (λ_ex_) of 290 nm. Control experiments were done with N-acetyl-Trp-amide (NATA) under the same conditions. Quenching of fluorescence intensities at the emission maximum (λ_em_) was observed by addition of increasing concentrations of potassium iodide, acrylamide, or succinimide (recrystallised), as appropriate. Stern-Volmer quenching constants (K_SV_) were determined by linear regression according to the standard equation:
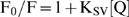
where F_0_ is the fluorescence emission intensity in the absence of quencher, and F is the observed intensity with quencher concentration [Q]. Fluorescence quenching of thermally- or chemically-denatured protein was studied under the same conditions using samples recovered from DSC experiments or fresh recombinant latherin dissolved in phosphate buffer containing 6 M guanidinium hydrochloride (GdnHCl, Invitrogen UltraPure™ grade), as appropriate. Putative hydrophobic ligand binding in solution was probed by comparison of ANS (ca. 10 µM) fluorescence emission (390 nm excitation) in the presence and absence of rlatherin.

### Neutron reflection

The adsorption of recombinant latherin at the air-water interface was investigated by neutron reflection, as previously described for other surfactant protein systems [42], [46]. Neutron reflectivity profiles were obtained using the SURF reflectometer (Rutherford Appleton Laboratory, ISIS, Didcot, UK) by established measurement procedures [35], [47]–[51] using a white beam source with neutron wavelengths in the range 0.5–6.5 Å. Samples were prepared by dissolving lyophilized protein (0.1 and 0.5 mg ml^−1^) in 11∶1 H_2_O/D_2_O to make null reflecting water (NRW) solution, or in D_2_O directly, and transferred to a Teflon trough mounted on an anti-vibration table in the reflectometer. As previously described [50], [51] experimental neutron reflectivity profiles were analyzed by means of the optical matrix modelling formalism in which the calculated reflectivity of an assumed layer model was compared with the measured data. The structural parameters were then varied by iteration in a least-squares procedure to give best fit. The structural parameters used in the fitting were the number of layers, thickness (τ), and the corresponding scattering length density (ρ) for each layer. The choice of the number of sublayers in the model depended upon the extent of inhomogeneity across the interface, but a single-layer model was sufficient to fit the data adequately here. These methods gave estimates of the thickness and volume fractions of protein layers at the air-water interface with depth resolution of order 1–3 Å along an axis normal to the plane of the interface.

## Supporting Information

Figure S1 Alignments of the four latherin sequences with the secondary structure predictions from SSPro 4.5; the program was used for this example because it does not rely on multiple alignments, although those programs that do use such alignments produced similar results for total secondary structure content (see below) and similar distribution of secondary structural elements (not shown). H = α-helix, E = β/extended strand, C = remainder.
(0.03 MB DOC)

Figure S2 Adsorption of recombinant latherin onto a waxy surface. (A) initial 10, 20, and 50 µl droplets of water, latherin and control protein (lysozyme), ca. 1 mg ml^−1^, on Nescofilm® sheet; (B) after blotting, photographed in oblique light to reveal residual film from latherin droplets; (C) after Coomassie Blue staining. Method: Small droplets (10, 20, 50 µl) of recombinant latherin solution, together with similar droplets of water and a non-surfactant control protein (hen egg white lysozyme, 1 mg ml^−1^ in water), were placed on the surface of strip of Nescofilm® sheet. After a few minutes, each drop was carefully blotted off using a tip of absorbent paper towel. The film was then stained for adsorbed protein by brief immersion in Coomassie Blue staining solution (BioRad), followed by rinsing with water. Each stage was photographed by digital camera. The scale is indicated by the centimetre rule. Result: Neither water nor control protein solution showed any evidence of residual surface wetting. However, as illustrated in Figure S2, after blotting, the latherin droplets left clear wet patches on the Nescofilm surface that subsequently stained positive for adsorbed protein. Separate experiments (not shown) confirmed that these surface layers were not permanent, but could be rinsed off easily with water prior to staining. This latter feature is presumably of functional significance, since permanent wetting would compromise the natural water-repellent properties of the oily pelt.
(3.54 MB DOC)

Table S1 Sequences of tryptic peptides obtained from horse sweat-derived latherin (LathPep1 to LathPep8).
(0.03 MB DOC)

Table S2 Oligonucleotide primers used in the study.
(0.03 MB DOC)
