# Supplementary material for: Latherin: A Surfactant Protein of Horse Sweat and Saliva
Source: PLoS One. 2009 May 29;4(5):e5726. doi: 10.1371/journal.pone.0005726 (PMC2684629; doi:10.1371/journal.pone.0005726)
Supplement: Figure S1 — Alignments of the four latherin sequences with the secondary structure predictions from SSPro 4.5; the program was used for this example because it does not rely on multiple alignments, although those programs that do use such alignments produced similar results for total secondary structure content (see below) and similar distribution of secondary structural elements (not shown). H = α-helix, E = β/extended strand, C = remainder. (0.03 MB DOC) [file pone.0005726.s001.doc]

**Latherin: a surfactant protein of horse sweat and saliva**

## SUPPORTING INFORMATION

**Figure S1.**

1 50

Horse MLKVSCLFVL LCGLLVPSSA QQIPPEVSSQ ITDALTQGLL DGNFLSLLNA

Onager .......... .......... QQIPPEVSSQ ITDALTQGLL DGNFLSLLNA

Ass .......... .......... QQIPPEVSSQ ITDALTQGLL DGNFLSLLNA

Zebra .......... .......... QQIPPEVSSQ ITDALTQGLL DGNFLSLLNA

Consensus .......... .......... QQIPPEVSSQ ITDALTQGLL DGNFLSLLNA

Secondary CCCCCCHHHH HHHHHHHHHC CHCHHHHHHH

51 100

Horse INLEGLLNTI LDQVTGLLNI LVGPLLGPSD AEIKLQDTRL LQLSLEFSPD

Onager INLEGLLNTI LDQVTGLLNI LVGPLLGPSN AEIKLQDARL LQLSLEFSPD

Ass INLEGLLNTI LDQVTGLLNI LVGPLLGPSN AEIKLQDARL LQLSLEFSPD

Zebra INLEGLLNTI LDQVTGLLNI LVGPLLGSSN AEIKLQDARL LQLSLEFSPD

Consensus INLEGLLNTI LDQVTGLLNI LVGPLLGpS# AEIKLQDaRL LQLSLEFSPD

Secondary HCCCCHHHHH HHHHHCHHHH CCCCCCCCCC EEEEECCHHH EEEEEECCCC

101 150

Horse SKGIDIWIPL ELSVYLKLLI LEPLTLYVRT DIRVQLRLES DEDGKYRLAF

Onager SKGIDIWIPL ELSVYLKLLI LEPLTLYVRT DIRVQLQLES DEDGKYRLAF

Ass SKGIDIWIPL ELSVYLKLLI LEPLTLYVRT NIRVQLQLES DEDGKYRLAF

Zebra SKGIDIWIPL ELSVYLKLLI LEPLTLYVRT DIRAQLQLES DEDGKYRLAF

Consensus SKGIDIWIPL ELSVYLKLLI LEPLTLYVRT #IRvQLqLES DEDGKYRLAF

Secondary CCCEEEEECC EHEEEEEEEE ECCCEEEEEC CEEEEEEEEC CCCCCEEEEE

151 200

Horse GHCSLLPRAI ELQSGNPLSL PVNAVLGTIE NALGNFITED LGAGLCPTLN

Onager GHCSLLPRAI ELQSGNPLSL TVNAVLGTIE NALGNFITED LGAELCPTLN

Ass GHCSLLPRAI ELQSGNPLSL TVNAVLGTIE NALGNFITED LGAELCPTLN

Zebra GHCTLLPRAI ELQTGNPLSL TVNAVLGTIE NTLGNFITED LGAGLCPTLN

Consensus GHCsLLPRAI ELQsGNPLSL tVNAVLGTIE NaLGNFITED LGAgLCPTLN

Secondary CCCCCCHHHE EEECCCCCCH HHHHHHHHHH HHHCHHHHHH HCCCCCHCHH

201 228

Horse SLVSNLDLQL VNNLINLILD RANVDLSV

Onager LLVSNLDLQL VNNLINLILD RANVDLSV

Ass SLVSNLDLQL VNNLINLILD RANVDLS.

Zebra SLVSNLNLQL VNNLINLILD RANVD...

Consensus sLVSNL#LQL VNNLINLILD RANVDls.

Secondary HHHHHHHHHH HHHHHHHHHH HCCCCCCC

Jpred : H regions: 29.3%, E regions: 27.4%

SSPro 4.5 2 class results: H regions: 38.9%, E regions: 23.1%

PredictProtein: H regions 34.62%, E regions 30.77%, remainder 34.62%
