# Supplementary material for: Latherin: A Surfactant Protein of Horse Sweat and Saliva
Source: PLoS One. 2009 May 29;4(5):e5726. doi: 10.1371/journal.pone.0005726 (PMC2684629; doi:10.1371/journal.pone.0005726)
Supplement: Figure S2 — Adsorption of recombinant latherin onto a waxy surface. (A) initial 10, 20, and 50 µl droplets of water, latherin and control protein (lysozyme), ca. 1 mg ml−1, on Nescofilm® sheet; (B) after blotting, photographed in oblique light to reveal residual film from latherin droplets; (C) after Coomassie Blue staining. Method: Small droplets (10, 20, 50 µl) of recombinant latherin solution, together with similar droplets of water and a non-surfactant control protein (hen egg white lysozyme, 1 mg ml−1 in water), were placed on the surface of strip of Nescofilm® sheet. After a few minutes, each drop was carefully blotted off using a tip of absorbent paper towel. The film was then stained for adsorbed protein by brief immersion in Coomassie Blue staining solution (BioRad), followed by rinsing with water. Each stage was photographed by digital camera. The scale is indicated by the centimetre rule. Result: Neither water nor control protein solution showed any evidence of residual surface wetting. However, as illustrated in Figure S2, after blotting, the latherin droplets left clear wet patches on the Nescofilm surface that subsequently stained positive for adsorbed protein. Separate experiments (not shown) confirmed that these surface layers were not permanent, but could be rinsed off easily with water prior to staining. This latter feature is presumably of functional significance, since permanent wetting would compromise the natural water-repellent properties of the oily pelt. (3.54 MB DOC) [file pone.0005726.s002.doc]

**Latherin: a surfactant protein of horse sweat and saliva**

## SUPPORTING INFORMATION

**Figure S2.**
