# Supplementary material for: Latherin: A Surfactant Protein of Horse Sweat and Saliva
Source: PLoS One. 2009 May 29;4(5):e5726. doi: 10.1371/journal.pone.0005726 (PMC2684629; doi:10.1371/journal.pone.0005726)
Supplement: Table S1 — Sequences of tryptic peptides obtained from horse sweat-derived latherin (LathPep1 to LathPep8). (0.03 MB DOC) [file pone.0005726.s003.doc]

**Latherin: a surfactant protein of horse sweat and saliva**

## SUPPORTING INFORMATION

**Table S1.** Sequences of tryptic peptides obtained from horse sweat-derived latherin (LathPep1 to LathPep8).

LathPep1 : LQDAR,

LathPep2 : LLQLSLEFSPDSK

LathPep3 : SIEIWIPLELSVYLKLLILEPLTLYVR

LathPep4 : AIEIWIPLELSVYLKLLILEPLTLYVR

LathPep5 : YRLAFGHDSLLPRAIELQSGNPLSLVVNAVLGQIENAL

LathPep6 : YRDAFGHDSLLPRAIELQSGNPLSLVVNAVLGQIENAL

LathPep7 : ANVDLS

LathPep8 : LAFGHDLLPR
