# Supplementary material for: Latherin: A Surfactant Protein of Horse Sweat and Saliva
Source: PLoS One. 2009 May 29;4(5):e5726. doi: 10.1371/journal.pone.0005726 (PMC2684629; doi:10.1371/journal.pone.0005726)
Supplement: Table S2 — Oligonucleotide primers used in the study. (0.03 MB DOC) [file pone.0005726.s004.doc]

**Latherin: a surfactant protein of horse sweat and saliva**

## SUPPORTING INFORMATION

**Table S2.** Oligonucleotide primers used in the study.

**1. Primers used to isolate partial cDNAs encoding horse latherin by RT-PCR.**

# Forward primers

# Horse F1A : ggg ccg tca gat gct gaa att aag ctt

Horse F2A : gac gct cga ctc ctt caa ctc tcc ctt

Horse F3A : ctc ctt att ctg gaa ccc ctg gcg cta

#### Reverse primers

Horse R1A : tct gac ata tag cgc cag ggg ttc

Horse R2A : cca tcc gtc gcc ccg gcg tgt caa

**Primers used to isolate cDNAs encoding latherin from other species**

Horse_Rev1 : tta gac gct gag gtc aac gtt cgc acg

Horse_Rev2 : aag tgt gag ggg ttc aag aat cag gag

**2. Primers used for the construction of recombinant protein expression vector**

5’ NcoI, 5'-ACCCTTCCATGGCTCAACAGATCCCACCTGAAGTTTCTTCC-3'

3’ Xho I, 5'-GGGAACTCGAGTTAGACGCTGAGGTCAACGTTCGC-3'

**3. Primers used to detect latherin-encoding transcripts by RT-PCR**

For latherin mRNA

Forward : 5’ CAA CAG ATC CCA CCT GAA GTT TCT TCC CAG 3’and

Reverse : 5’ TTA GAC GCT GAG GTC AAC GTT CGC 3’,

For ribosomal S15 protein mRNA

Forward : 5’ TTC CGC AAG TTC ACC TAC C 3’

Reverse : 5’ CGG GCC GGC CAT GCT TTA CG 3’.
